# A Practical Treatise on Bright’s Diseases of the Kidneys

**Published:** 1872-03

**Authors:** 


*A Practical Treatise on Bright's Diseases of the Kidneys.* By T. GRAINGER STEWART, M. D., F. R. S. E. New York, Wm. Wood & Co., 1871.

Having added to his stock of knowledge, by three years of observation in this department of Medical Investigation, Dr. Stewart hastens to place it before the medical world, in the form of a revision of his excellent work on Bright's Diseases. The book before us is written in a plain and concise manner, and bears evidence of coming from the pen of a man who knows "whereof he speaks."

The work is illustrated by nine well executed plates. Beside the regular consideration of the subject, three subjects of special interest are treated of in supplementary chapters. The profession are under many obligations to Dr. Stewart, for his interesting and valuable work.

